# Two-Step Solid-State Synthesis of Ternary Nitride Materials

**DOI:** 10.1021/acsmaterialslett.1c00656

**Published:** 2021-11-02

**Authors:** Paul K. Todd, M. Jewels Fallon, James R. Neilson, Andriy Zakutayev

**Affiliations:** † Material Science Center, National Renewable Energy Laboratory, Golden, Colorado 80401, United States; ‡ Department of Chemistry, Colorado State University, Fort Collins, Colorado 80523-1872, United States

## Abstract

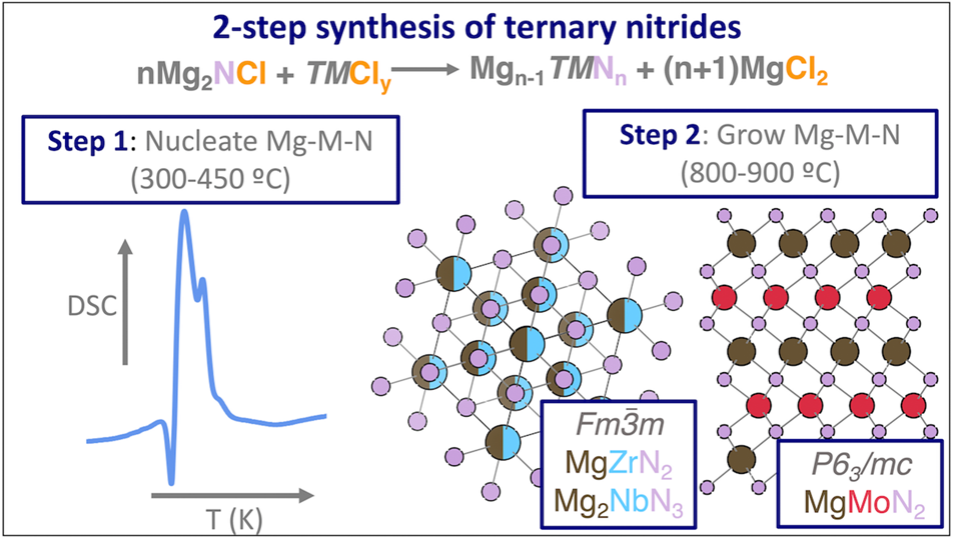

Ternary nitride materials hold promise for many optical, electronic, and refractory applications; yet, their preparation via solid-state synthesis remains challenging. Often, high pressures or reactive gases are used to manipulate the effective chemical potential of nitrogen, yet these strategies require specialized equipment. Here, we report on a simple two-step synthesis using ion-exchange reactions that yield rocksalt-derived MgZrN_2_ and Mg_2_NbN_3_, as well as layered MgMoN_2_. All three compounds show almost temperature-independent and weak paramagnetic responses to an applied magnetic field at cryogenic temperatures, indicating phase-pure products. The key to synthesizing these ternary materials is an initial low-temperature step (300–450 °C) to promote Mg-M-N nucleation. The intermediates then are annealed (800–900 °C) to grow crystalline domains of the ternary product. Calorimetry experiments reveal that initial reaction temperatures are determined by phase transitions of reaction precursors, whereas heating directly to high temperatures results in decomposition. These two-step reactions provide a rational guide to material discovery of other bulk ternary nitrides.

Ternary metal nitrides remain under-explored as new functional inorganic materials,^1,2^ even though a large number of new nitride compositions and structure types have been recently predicted.^3−6^ The deficit in realized nitride products, compared to predicted materials, stems from their difficult synthesis, with few successful reactions that yield nitride products selectively.^7^ Furthermore, control over composition is limited when attempting to synthesize nitrogen-rich nitride semiconductors^8^ and mechanically ultrahard pernitrides,^9^ which have a tendency to be metastable, when compared to their metallic subnitride analogues.^10^ To access more nitrogen-rich phases, reactions must proceed at low temperatures, where dinitrogen (N_2_) formation is less thermodynamically favorable, or higher temperature reactions must change the effective chemical potential within the reaction system through use of high pressures or reactive gases, such as ammonia. Furthermore, a large number of potential binary metal nitride precursors are either refractory^11,12^ or energetic,^13−17^ which further reduces the number of useful reactions. Therefore, identifying sources of reactive nitrogen that yield desired products under mild conditions is imperative for advancement in nitride material discovery.

Various reactive nitrogen sources are used for ternary metal nitride synthesis. In bulk form, ternary nitrides have been synthesized by high-pressure metathesis,^18,19^ ammonolysis,^20^ ammonothermally,^21^ within alkali-metal fluxes,^22^ self-combustion,^23^ and rarely from the elements under flowing N_2_.^24^ For thin film materials discovery and applications, an excited nitrogen plasma can be employed to deposit ternary metal nitrides ranging from rocksalt magnesium metal nitrides, such as MgZrN_2_ and Mg_2_NbN_3_,^25,26^ to wurtzite zinc metal nitrides, such as Zn_2_SbN_3_ and Zn_3_MoN_4_.^27,28^ For the ambient-pressure synthesis of bulk magnesium metal nitrides, precursors that react at low temperatures must be selected, since the loss of volatile elements, such as nitrogen, magnesium, or zinc, from the ternary product occurs at high temperatures. As a counterexample, reactions using Mg_3_N_2_ and refractory transition metals, such as zirconium or molybdenum, will not proceed before Mg_3_N_2_ decomposes, while metathetical preparations between Mg_3_N_2_ and transition-metal halides^29^ result in binary metal nitrides or reduced metals. To overcome these challenges, high-pressure autoclaves are used in conjunction with sodium azide at 700 °C, as seen in the synthesis of crystalline MgMoN_2_.^30^

As a gentler alternative to high-pressure synthesis, mixed-anion magnesium chloride-nitride (Mg_2_NCl) has been recently used for lowering reaction temperatures in the preparation of binary Mn_3_N_2_^31^ and ternary Mg_*x*_Zr_1–*x*_N.^32^ When compared to reactions starting with Mg_3_N_2_, Mg_2_NCl results in more magnesium inclusion into the ternary product, as well as faster reaction times. While MgZrN_2_ was reported, optical and electronic property measurements exhibit significant metallic behavior attributed to ZrN nanodomains, compared to previous reports of semiconducting thin film samples,^26,33^ calling for improved control of the bulk synthesis conditions and reaction pathways. Furthermore, analysis of reaction thermodynamics starting from Mg_2_NCl^34^ and yielding MgZrN_2_^32^ suggests that the reaction pathways can be controlled through careful precursor selection for other ternary nitride compositions.

Here, we describe the synthesis of three magnesium metal nitrides, where a transition-metal halide (ZrCl_4_, NbCl_5_, MoCl_5_) reacts with magnesium chloride-nitride to yield each magnesium metal nitride product (MgZrN_2_, Mg_2_NbN_3_, MgMoN_2_) and equivalent amounts of MgCl_2_ byproduct:
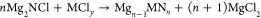
1These ternary metal nitrides are synthesized close to ambient pressure via two-step reactions, where precursors are first heated at relatively low temperature (300–450 °C) to promote Mg–M–N nucleation, and then the temperature is increased (800–900 °C) to grow crystalline domains of the ternary metal nitride products. Differential scanning calorimetry (DSC) experiments reveal exothermic events that occur near the first low-temperature step for each composition, indicating intermediate reactions that likely yield magnesium metal nitride products. As a result of these two-step reactions, MgZrN_2_ and Mg_2_NbN_3_ are observed in a rocksalt-derived structure while MgMoN_2_ adopts a layered hexagonal structure. The ternary nitride products have close to stoichiometric cation compositions which are consistent with their weak paramagnetic behavior, as opposed to a strong diamagnetic response characteristic of the binary nitride impurities. These results demonstrate a low-temperature two-step solid-state synthesis approach to ternary nitride materials.

For each ternary nitride synthesis performed at NREL, homogeneously mixed precursor powders were pelletized under argon and flame-sealed in evacuated quartz ampules. For reactions yielding MgZrN_2_ and Mg_2_NbN_3_, ampules were heated in a muffle furnace to 450 °C for 24 h, followed by a subsequent anneal at 800 °C for 24 h. Similarly, reactions yielding MgMoN_2_ were heated at 300 °C for 24 h, then at 900 °C for 24 h. Cation compositions were measured using energy-dispersive X-ray spectroscopy (EDX). Powder X-ray diffraction (PXRD) was used to characterize each product’s crystal structure and bulk magnetic susceptibility measurements using vibrating sample magnetometry (VSM) confirmed the product composition and purity. Temperature-dependent reaction profiles were determined from DSC experiments. More detailed accounts of synthesis methods and characterization techniques are provided in the Supporting Information.

Using these two-step metathesis reactions, three magnesium metal nitrides were selectively prepared and confirmed through diffraction. Figure 1a depicts PXRD patterns of the reaction products—MgZrN_2_, Mg_2_NbN_3_, and MgMoN_2_—after washing with anhydrous methanol to remove MgCl_2_ products. Quantitative crystallographic analysis using the Rietveld method reveals that the MgZrN_2_ and Mg_2_NbN_3_ crystallize in the rocksalt (*Fm*3̅*m*) structure, as previously reported in thin-film products,^25^ whereas MgMoN_2_ forms in the layered hexagonal crystal structure (*P*6_3_/*mc*), as previously reported in bulk form, and^24^ as illustrated in Figure 1c. The simulated XRD patterns are shown in Figure 1b for comparison, while structural parameters for each product are listed in Table 1 and compared to literature values.

**Figure 1:**
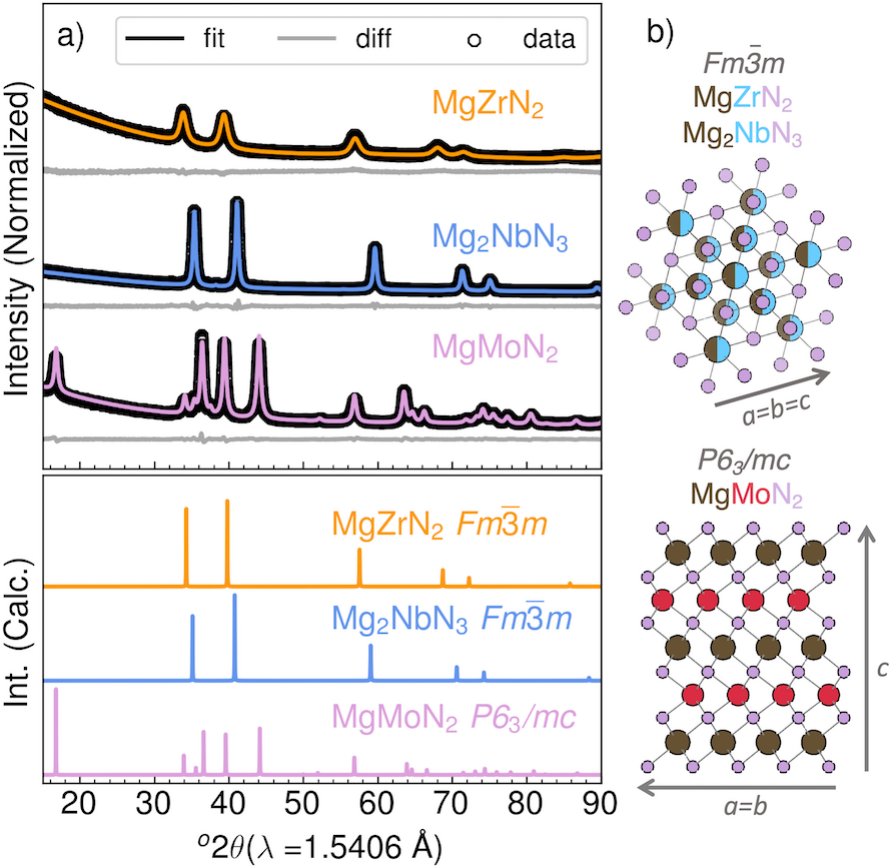
Crystallographic analysis of three magnesium metal nitride products. (a) Rietveld refinements of PXRD patterns of magnesium metal nitride products along with the simulated reference diffraction patterns and space group of each structure. (b) Pictorial representations of the cubic rocksalt-derived structure for MgZrN_2_ and Mg_2_NbN_3_, and a layered hexagonal crystal structure for MgMoN_2_.

**Table 1:** Refined Structural Parameters for the Magnesium Metal Nitride Products,^a^ Compared to Reference Values and to Literature Data, and Phase Fraction of Binary Metal Nitride Impurity, as Measured from PXRD and VSM Measurements

	Structure (PXRD)			Composition, *x* = Mg/(Mg + M)		Binary Nitride Phase Fraction	
compound	*a* = *b* (Å)	*c* (Å)	*V* (Å^3^)	EDX^b^	PXRD	PXRD (mol % M_*x*_N_*y*_)	VSM (vol % SC M_*x*_N_*y*_)
MgZrN_2_	(*Fm*3̅*m*)			0.50	0.50	0	0
present	4.541(2)	–	93.67(1)	0.40	0.48(8)	–	2.2 × 10^–3^
ref (25)	4.54	–	–	0.49	–	–	–
							
Mg_2_NbN_3_	(*Fm*3̅*m*)			0.67	0.67	0	0
present	4.386(3)	–	84.39(2)	0.64	0.60(6)	–	8.13 × 10^–3^
ref (25)	4.37	–	–	0.68	–	–	–
							
MgMoN_2_	(*P*6_3_/*mc*)			0.50	0.50	0	0
present	2.924(1)	10.4716(6)	77.54(6)	0.46	0.53(4)	0.53(7)	1.8 × 10^–3^
ref (24)	2.91059(3)	10.5484(1)	–	0.52	0.48	–	–

a Measured chemical composition: *x* = Mg/(Mg + M) in Mg_*x*_M_1–*x*_N_*y*_.

b EDX errors range from 5% to 10%.

For the two rocksalt structures, MgZrN_2_ and Mg_2_NbN_3_, the observed PXRD patterns support magnesium inclusion into the rocksalt structure by a change in the (111) peak intensity, which is indicative of less electron density of the magnesium cation. Rietveld analysis permits refinement of the site occupancies of the 4a Wykoff position in the rocksalt structure, which accounts for the change in relative peak intensity in Figure 1a, with the *x* = Mg/(Mg + M) values reported in Table 1. For Mg_*x*_Zr_1–*x*_N_*y*_ and Mg_*x*_Nb_1–*x*_N_*y*_, the cation concentrations fall within the limits that have been previously reported for these cation-disordered solid solutions.^25,26,32^ However, Mg_*x*_Zr_1–*x*_N_*y*_ (*x* = 0.48) and Mg_*x*_Nb_1–*x*_N_*y*_ (*x* = 0.60) reported here reveal a deficiency of magnesium, compared to ideal values (*x* = 0.50 and *x* = 0.67, respectively). Furthermore, the peak width for these rocksalt phases broadens with increasing cation site disorder, which has been previously observed for the solid-solution Mg_*x*_Zr_1–*x*_N.^32^

Fitting the layered hexagonal structure of MgMoN_2_ using Rietveld refinement reveals an absence in intensity in the (0 0 *l*) family of reflections, relative to peaks associated with atoms in the (*h* 0 1), as similarly observed for structurally analogous MnMoN_2_.^35^ This observation can be explained by either disorder in the (0 0 1) direction of the MgMoN_2_ layers or preferred orientation of crystallites in the (1 0 1) direction. During the Rietveld analysis, applying preferred orientation in the (1 0 1) direction accounts for the increase in intensity of these reflections, relative to the (1 0 *l*) family of peaks. Furthermore, there is a contraction of the *c*-axis (Table 1), which could indicate the presence of smaller Mo^5+^ cations in the nominally Mo^4+^ site, likely due excess magnesium incorporation. Free refinement of each cation site in the *P*6_3_/*mc* lattice (Table 1) supports greater magnesium content than molybdenum in Mg_*x*_Mo_1–*x*_N_*y*_ (refined *x* = 0.53, compared to *x* = 0.50 reference value), along with some cation deficiency on the molybdenum site with (Mg + Mo)/(Mg + Mo + N) = 0.85, compared to the 1.00 reference value.

The relative cation composition in these Mg_*x*_Zr_1–*x*_N_*y*_, Mg_*x*_Nb_1–*x*_N_*y*_ and Mg_*x*_Mo_1–*x*_N_*y*_ materials, where *x* = Mg/(Mg + M), was confirmed by EDX analysis. For these metals, the EDX peak intensities are high enough to provide reasonable error. The nitrogen and oxygen differentiation is not as facile, because of low signal-to-noise ratio in the low-energy part of the spectrum, as well as high background oxygen counts from the substrate. As presented in Table 1, the EDX results show magnesium and transition-metal compositions that fall within the limits determined from XRD refinement and previously reported in other publications,^24−26,32^ although, for the rocksalt products, this ratio is substoichiometric, with regard to magnesium. Therefore, it is likely that some magnesium is lost during the reaction, because of the thermal decomposition of Mg_2_NCl at higher temperatures, despite intentional excess of this precursor in the reactions. In all reactions, a metal deposit is present on the quartz ampule, supporting the reduction of magnesium and the formation of N_2_.

To further evaluate the phase purity of our Mg-M-N products, magnetic susceptibility measurements were performed at CSU. The results in Figure 2 exhibit weak paramagnetic behavior (χ > 0), which supports the compositions presented in Table 1. For each of these ternary Mg-M-N products, a binary metal nitride or oxynitride impurity (ZrN, NbN, Mo_2_N) should produce a diamagnetic response from a superconducting transition, which is virtually absent in Figure 2 for the samples reported in Table 1. To illustrate the effect of even small fractions of binary nitride impurities, reaction products were treated with 1 M nitric acid in attempts to leach out magnesium. These leaching experiments (Figure 2) lead to a clear decrease in the magnetic susceptibility at low temperatures, which is indicative of a superconducting transition in the binary impurity. Note that the pure products washed with dry methanol (Figure 2 and Table 1) do exhibit a very small superconducting transition (Figure S1 in the Supporting Information) corresponding to <0.001 vol % impurity; yet, these values are significantly lower in superconducting phase fraction than the products leached with nitric acid.

**Figure 2:**
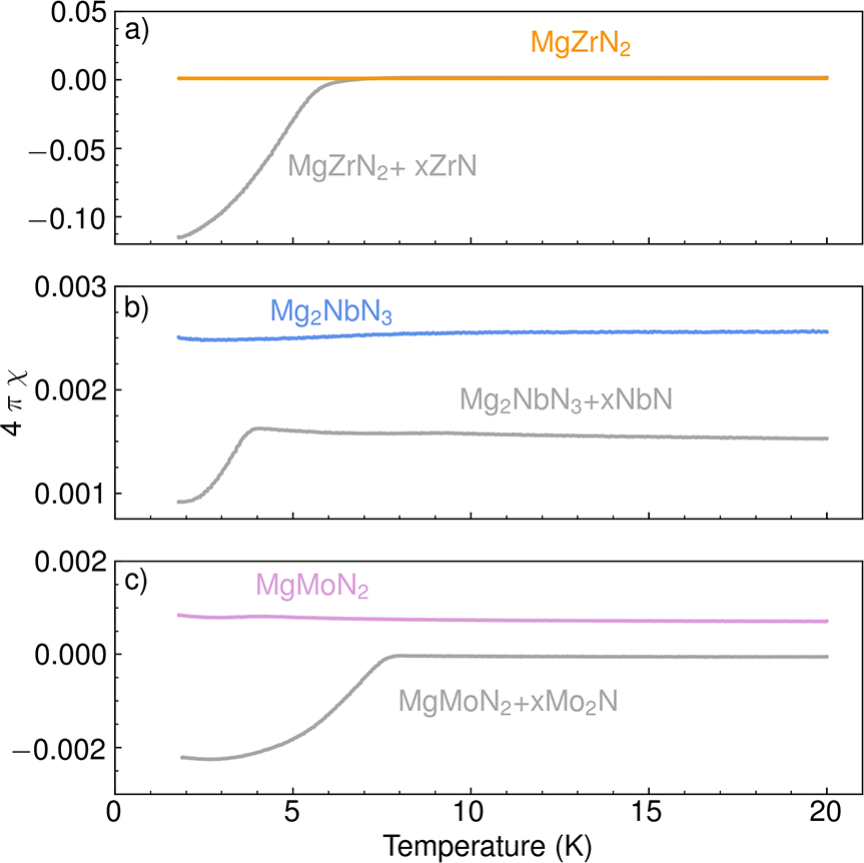
Temperature-dependent magnetic susceptibility of magnesium metal nitride ternary products: (a) MgZrN_2_, (b) Mg_2_NbN_3_, and (c) MgMoN_2_. The phase-pure products washed with dry methanol are shown in contrast to samples with magnesium leached out by 1 M nitric acid, leading to binary transition-metal nitride impurities with clear superconducting transitions. DC magnetization data were collected using a measurement field of *H* = 20 Oe.

The described synthesis conditions in eq 1 require two-step temperature profiles where an initial temperature (*T*_nuc_) nucleates metal nitride products, followed by a higher crystallite growth temperature (*T*_gr_). Figure S2 in the Supporting Information depicts PXRD patterns of the unwashed products observed when using these two-step heating profiles, compared to directly heating to *T*_gr_. When heated directly to 800 °C, the rocksalt MgZrN_2_ product observes a clear shift in lattice parameter (see Figure S2a in the Supporting Information) toward ZrN paired with an increase in the relative intensity of the (1 1 1) peak, supporting a loss of Mg. For Mg_2_NbN_3_ products, heating directly to 800 °C results in broad peaks in the PXRD pattern (Figure S2b in the Supporting Information) with a shift toward a smaller lattice parameters than the reaction product via two-step heating schedule. The calculated ground-state lattice parameter of 4.42 Å is larger than that of binary NbN, yet thin-film Mg_2_NbN_3_ reports a lattice parameter of 4.37 Å.^25^ For reactions yielding MgMoN_2_, directly heating above 800 °C yields more Mo_2_N than MgMoN_2_, whereas the described two-step heating profile increases the yield of MgMoN_2_, as seen in Figure S2c in the Supporting Information.

To gain insight into the low-temperature reaction pathway, we performed DSC experiments presented in Figure 3. These DSC results reveal new low-temperature exothermic reactions paired with known endothermic phase transitions of the respective transition-metal halide precursors. Mg_2_NCl does not have a phase transition below 600 °C, suggesting that observed exotherms are attributed to the formation of MgCl_2_, Mg-M-N products, or unknown intermediate species. For the reactions yielding MgZrN_2_ (Figure 3a), there is an exotherm observed after the sublimation temperature of ZrCl_4_ at 331 °C (Zr1: 366 °C). At 411 °C, a large endothermic inflection is observed, which we attribute to the pressure-induced melting of ZrCl_4_ from the gaseous state near 437 °C.^36^ For reactions yielding Mg_2_NbN_3_ (Figure 3b), a similar exothermic peak is observed after the melting point of NbCl_5_ at 205 °C (Nb1: 208 °C; Nb2: 216 °C), with two additional exotherms—Nb3: 450 °C and Nb4: 513 °C—also observed. For the MgMoN_2_ reaction in Figure 3c), no phase transition endotherm is observed for MoCl_5_ at the expected melting point of 194 °C, yet a triplet of exothermic peaks is observed near this transition temperature (Mo1: 174 °C, Mo2: 200 °C, and Mo3: 233 °C). Furthermore, there are two additional broad exotherms at higher temperatures (Mo4: 465 °C and Mo5: 550 °C).

**Figure 3:**
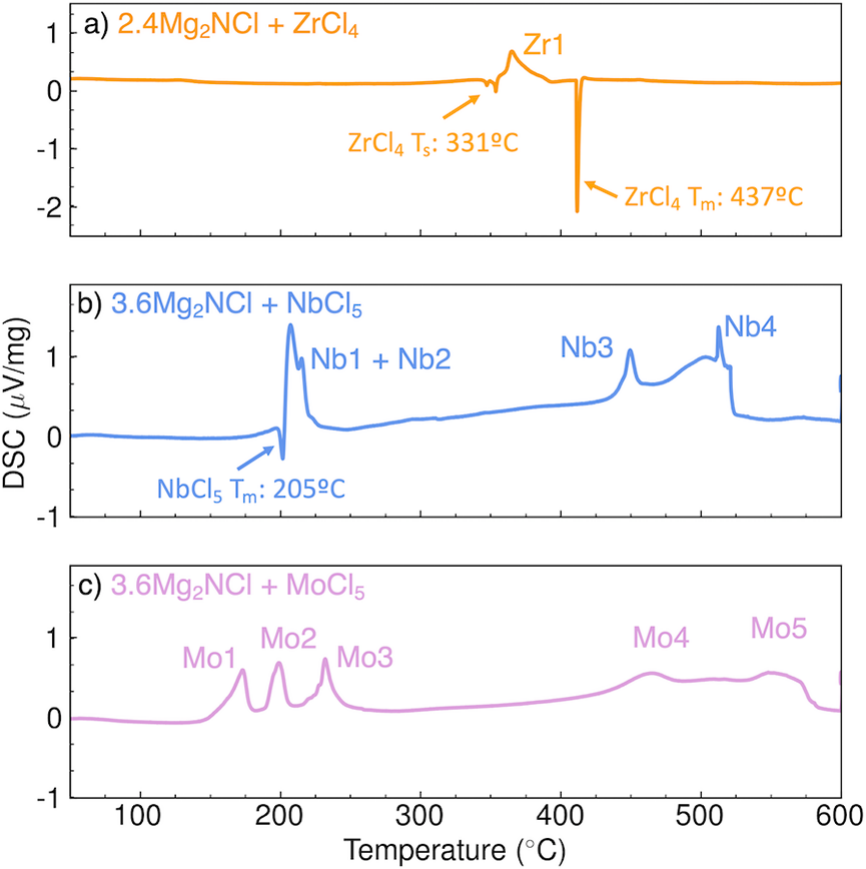
Synthesis reaction pathway from DSC measurements for (a) MgZrN_2_, (b) Mg_2_NbN_3_, and (c) MgMoN_2_. The endotherms correlate with transitions of metal halide precursors and the exotherms correspond to the nucleation of ternary products.

Using the measured temperatures of relevant exothermic peaks in Figure 3, control reactions were performed, targeting three *T*_nuc_ values (300, 450, and 600 °C) and two *T*_gr_ values (800 and 900 °C) to evaluate the effect of temperature on the reaction products. Figure S4a in the Supporting Information depict the changes in hexagonal lattice parameters, as a function of heating schedule for MgMoN_2_ products. Here, the proposed *T*_nuc_ of 300 °C yields lattice parameters most similar to MgMoN_2_.^24^ As *T*_nuc_ increases, the *a*-axis lattice parameter remains constant, whereas the *c*-axis lattice parameter decreases. In addition, Figure S4a further supports increased MgMoN_2_ yields at a lower *T*_nuc_ values, whereas higher initial temperatures result in greater Mo_2_N yields. Contrary to the lower *T*_gr_ value of the rocksalt-yielding reactions, MgMoN_2_ product yields increase at 900 °C, albeit only with a two-step temperature profile.

For rocksalt MgZrN_2_ and Mg_2_NbN_3_ products from these control reactions, Rietveld analysis was used to quantify changes in lattice parameter (Figure S4a), as well as the changes in peak intensity relative to the (1 1 1) by allowing the magnesium:transition-metal ratio to openly refine (see Figure S3). For MgZrN_2_, the proposed heating schedule results in the smallest lattice parameter and largest Mg concentration in Figure S5a in the Supporting Information. In addition, the lower *T*_gr_ results in peak broadening, as calculated in Figure S5b in the Supporting Information, which also supports increased cation disorder in these rocksalt structures. For Mg_2_NbN_3_ the change in lattice parameter in Figure S5a is less indicative of increased Mg content; yet, changes in peak shape in Figure S5b support a similar trend of increased magnesium content with broadened peak shape, which requires a lower *T*_gr_ value.

The collective results presented in this letter reveal a synthesis approach to ternary magnesium metal nitrides at low temperatures and ambient pressures (Figure 1). A key to this two-step process is the dependence on a low-temperature reaction *T*_nuc_. We suggest that *T*_nuc_ yields Mg-*M*-N nucleation, as evidenced by the numerous exothermic events from DSC (Figure 3), and the absence of ternary products from direct heating (Figure S2 in the Supporting Information). This low-temperature reaction step at *T*_nuc_ ensures that solid-state diffusion can proceed below the temperature where product decomposition is observed, because of the overall small changes in formation energies and the increasing entropic driving force for N_2_ formation.^5,10^ This low-temperature reaction pathway is facilitated by the low melting points of the transition-metal halide precursors, according to the DSC measurements in Figure 3. These transition-metal halides form monomeric or dimeric species as they melt,^37−39^ which reduces diffusion lengths at the reaction interface, thus ensuring that necessary ion exchange yields Mg-M-N intermediate phases or poorly crystalline products. Heating to higher *T*_gr_ temperatures too quickly results in deleterious sublimation and decomposition of these halide precursors. Thus, a higher *T*_gr_ temperature may be required to increase the crystallinity of the products, yet the *T*_nuc_ reaction temperature is the most likely “rate-limiting” step in this two-step reaction pathway.

The presented reaction conditions are benign and can be performed in a traditional solid-state chemistry laboratory, thus increasing their utility in targeting other metal nitride compositions by multiple research groups. Previous studies on the synthesis of magnesium metal nitrides have employed custom high-pressure reactors,^18,19^ or specialized deposition chambers.^25,26^ By starting with the mixed anion Mg_2_NCl as a precursor, the reaction pathway does not proceed via a rapid propagation, as observed when starting with more energetic precursors, such as alkali azides or alkali-earth nitrides.^13,40^ Furthermore, diffusion-limited products and binary metal nitrides observed in some metathesis reactions^40,41^ are avoided. The presented reactions avoid toxic environments, such as ammonia or amide-based mineralizers,^21,42,43^ that decompose under elevated temperatures and require careful safety considerations and custom equipment. Similar to the abundance of metal halide precursors, numerous metal chloride-nitride phases exist and are easily synthesized.^44−46^ For example, Zn_2_NX (X = Cl, Br, I) precursors may provide a low-temperature route to zinc metal nitrides, such as Zn_2_NbN_3_ and ZnZrN_2_, which exhibit a loss of zinc at elevated temperatures.^47−50^

The degree of precursor interchangeability in eq 1 provides a design space for discovering new ternary metal nitrides and controlling their properties. Optoelectronic property measurements on bulk^32^ and thin-film^26,33^ Mg_1–*x*_Zr_*x*_N_2_ samples exhibit tunable metal to semiconductor electronic properties as the magnesium content increases with band gaps and effective masses that exhibit remarkable tolerance to structural disorder.^25^ Similar behavior has also been observed in Mg_2–*x*_Nb_1+*x*_N_3_ thin films,^25^ while MgMoN_2_ is calculated to be metallic.^51^ Bulk MgZrN_2_, Mg_2_NbN_3_, and MgMoN_2_ materials reported here are expected to exhibit similar optoelectronic properties based on the stoichiometric composition range observed (Table 1) and the absence of binary metal nitride impurities (Figure 2). As a next step, synthesizing new zinc metal nitride compositions, as well as potential Mg/Zn alloyed quaternary phases could lead to precise tuning of these optoelectronic properties.

In summary, we report on the bulk solid-state synthesis of three magnesium metal nitrides—MgMoN_2_ with layered hexagonal structure, and MgZrN_2_, Mg_2_NbN_3_ with rocksalt-derived structure—using two-step low-temperature ion-exchange reactions. An initial low-temperature reaction of the precursors nucleates the magnesium metal nitride product, and that is followed by a high-temperature step to grow the product crystalline domains. The products are measured by PXRD, with cation stoichiometry confirmed by EDX, and phase purity supported by magnetic susceptibility measurements. Characterizing this reaction pathway using DSC reveals multistep crystallization that occurs at low temperatures, which we attribute to the formation of an intermediate ternary product with short-range ternary metal nitride bonds but without long-range crystallographic order. In contrast, by heating the precursors too rapidly, before they can successfully nucleate the magnesium metal nitride products, results in a net loss of Mg and N at high temperature. The results presented here indicate that this low-temperature ambient-pressure approach can be used to synthesize other ternary nitride materials.

## Supporting Information Available

Experimental methods and additional results from control reactions (PDF)
