# Supplementary material for: Two-Step Solid-State Synthesis of Ternary Nitride Materials
Source: ACS Mater Lett. 2021 Nov 2;3(12):1677–83. doi: 10.1021/acsmaterialslett.1c00656 (PMC10961828; doi:10.1021/acsmaterialslett.1c00656)
Supplement: Supplementary file 1 — tz1c00656_si_001.pdf [file tz1c00656_si_001.pdf]

Supplementary Information: Two-step solid-state synthesis of ternary nitride materials

Paul K. Todd,*,† M. Jewels Fallon,‡ James R. Neilson,*,‡ and Andriy Zakutayev*,†

†*National Renewable Energy Laboratory, Materials Science Center, Golden, Colorado 80401, United States*

‡*Colorado State University, Department of Chemistry, Fort Collins, Colorado 80523, United States*

E-mail: Paul.Todd@nrel.gov; James.Neilson@colostate.edu; Andriy.Zakutayev@nrel.gov

1 Supplementary Methods

1.1 Precursor Preparation

All reagents were prepared and stored in an argon-filled glovebox where O_2 and H_2O levels were $\leq$ 0.1 ppm. Molybdenum (V) chloride (99.99%, Sigma Aldrich), Niobium (V) chloride (99.995%, Sigma Aldrich), Zirconium (IV) chloride (99.99%, Sigma Aldrich) were purchased and stored under argon. Magnesium nitride chloride was prepared by mixing anhydrous magnesium chloride (99.99% Sigma Aldrich) and anhydrous magnesium nitride (99.5%, Sigma Aldrich) in a 1:1 molar ratio and homogenizing in an agate mortar in pestle (15 min). The homogenized mixture was pelleted (1/2 in diameter) at (100 bar) and sealed in a 14 mm by 16 mm (IDxOD) quartz ampule under vacuum ($\leq$ 15 mTorr). To transfer the ampule from the argon-filled glovebox to the vacuum sealing line, the quartz ampule was connected via an o-ring sealed quick connect fitting attached to a butterfly valve. A methane-oxygen torch was used to seal a 13 mm outer diameter quartz plug and the reaction ampule. This sealed ampule was then placed in a muffle furnace and heated to 550 °C at 3 °C/min for one week.

1.2 Material Synthesis

For the synthesis of magnesium metal nitrides, the prepared Mg_2NCl and corresponding metal halides were homogenized in a mortar and pestle (15min). The homogenized powders were pressed (1/4 in diameter) into a pellet (100 bar) and sealed in a 10mm by 12 (IDxOD) quartz ampule using a 9mm quartz plug using the same technique described above for Mg_2NCl. The sealed ampules were placed into a muffle furnace and heated according to the following heating schedules. For magnesium zirconium nitride and magnesium niobium nitride, the reaction was heated at 10 °C/min to 450 °C for a 24 h dwell and subsequently heated to 800 °C for an additional 24 h anneal. For magnesium molybdenum nitride, the reactions were heated at 10 °C/min to 300 °C for a 24 h dwell and subsequently heated to 900 °C for an additional 24 h anneal. After the final anneal, all reactions were allowed to cool to 25 °C by simply turning off the furnaces.

1.3 Material Characterization

Powder X-ray diffraction (PXRD) was performed on a Rigaku Ultima IV diffractometer using Cu Kα radiation. Samples were collected on an amorphous silicon wafer to reduce background interference. To avoid air exposure for some PXRD samples, polyimide tape was placed over the top of the sample. Rietveld analysis was perfomed in TOPAS. Peak shape was calculated using a Psuedo-Voigt approximation of size and strain. Differential Scanning Calorimetry (DSC) experiments were conducted on a Netzsche DSC 404 F3 Pegasus. Samples were prepared by loading precursors into an aluminum pan and sealed in a glovebox using a hermetic sealing tool. DSC experiments were heated at 5 °C/min to 600 °C, which is just below the limit of the aluminum pans. SEM-EDX was performed on a Hitachi 4800 SEM with a Thermo Fisher Pathfinder EDX detector. Samples were mounted on double sided carbon tape. EDX was conducted at a 15mm working distance to maximize take off angle and collection of any X-rays emitted from samples. EDX conditions were set at a minimum of 10 keV energy and 10 μA.

A Quantum Design Magnetic Properties Measurement System (MPMS3) was used for measuring samples. Samples were prepared by weighing about 10-15 mg of product into a VSM powder sample holder. Air-free samples were prepared in the glovebox and eicosane (Acros Organics, 99 %) was placed on top of the samples and melted upon removal from the glovebox to help keep the samples air-free. The zero field-cooled magnetization was measured from 1.9 K to 20 K at a field strength of H = 20 Oe. The magnetization was converted to volume susceptability using field strength, mass, and the calculated density of ρ = 4.41 g cm^{-3} for Mg_2NbN_3, ρ = 6.36 g cm^{-3} for $MgMoN_2$ and ρ = 5.09 g cm^{-3} for $MgZrN_2$.

2 Supplementary Results

Table S1: Magnetic Susceptibility data of mangesium metal nitride products washed with different solvents.

Compound	Purification	T_c	vol % SC M_xN_y
$MgZrN_2$	MeOH-an	~ 9.5	0.0022
	MeOH	8.5	4.84
	1M HNO_3	6.5	11.87
Mg_2NbN_3	MeOH-an	11.5	0.0082
	MeOH	N/A	0
	1M HNO_3	4	0.0063
$MgMoN_2$	MeOH-an	~ 4.5	0.0018
	MeOH	3.5	0.0198
	1M HNO_3	8	0.225

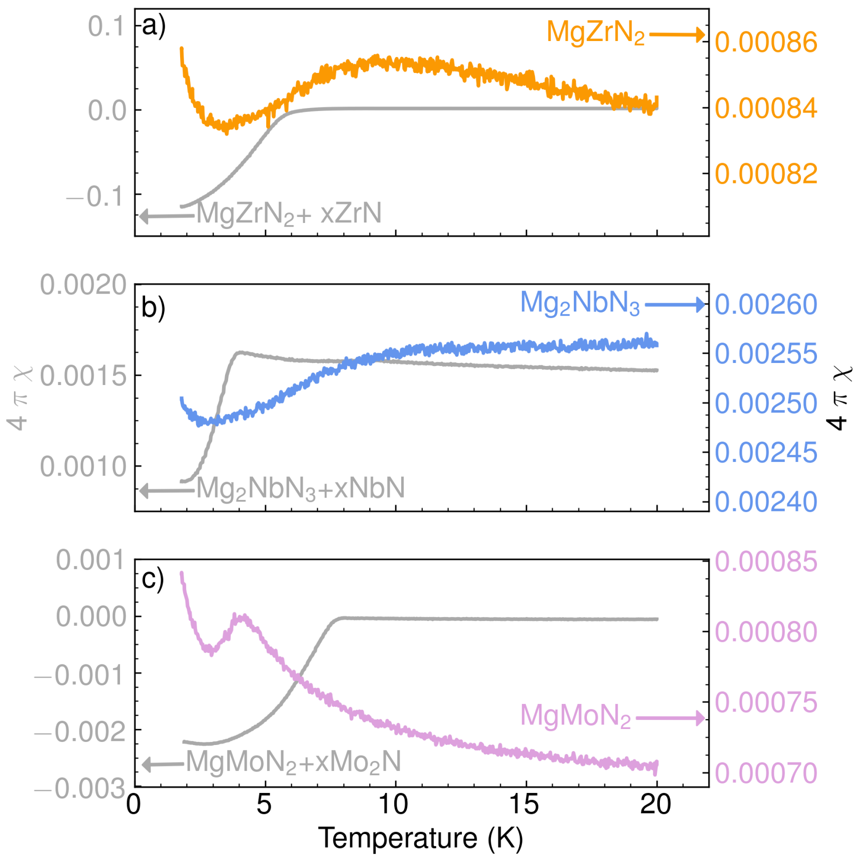

Figure S1: Temperature dependent magnetic susceptibility of magnesium metal nitride ternary products: a)$MgZrN_2$, b) Mg_2NbN_3, c) $MgMoN_2$. All measurements were conducted at 20 Oe. Nominally phase pure products washed with dry methanol are shown in contrast to samples washed with 1M nitric acid, which leads to magnesium dissolution. Superconducting transitions are observed due to binary nitride or oxynitrides. Here, each measurement is scaled for visualization purposes with arrows indicating which axes corresponds to which measurement.

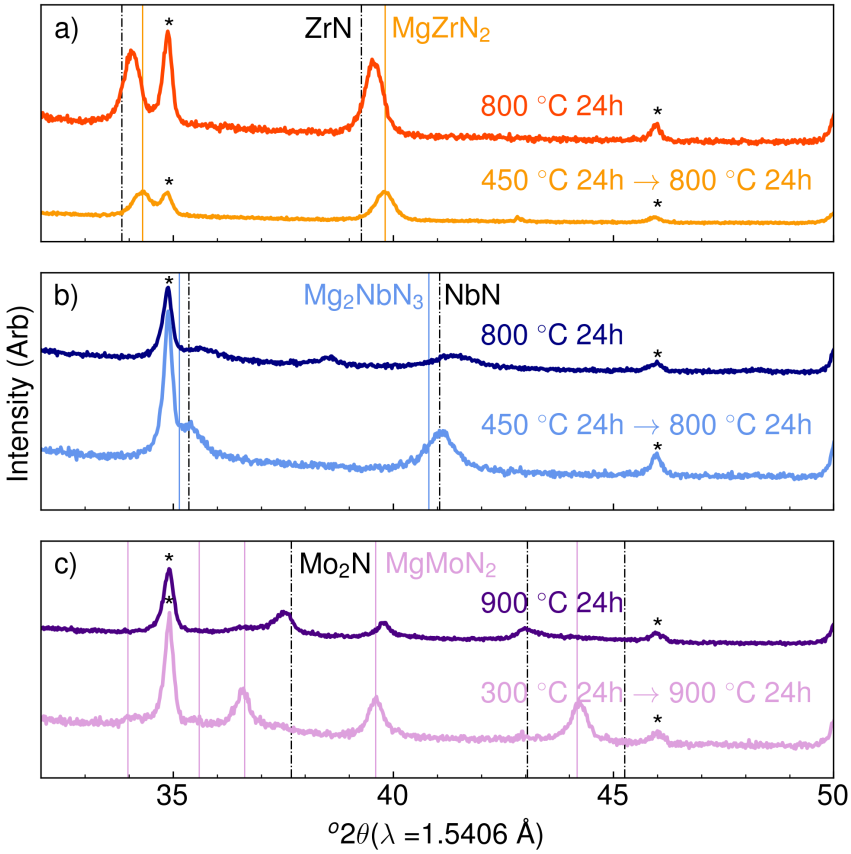

Figure S2: Powder X-ray diffraction patterns of products from the reaction: $n\mathrm{Mg_2NCl} + \mathrm{MCl_y} \longrightarrow \mathrm{Mg_{n\text{-}1}MN_n} + n\text{+}1\mathrm{MgCl_2}$, where M = a) Zr, b) Nb, c) Mo). For each reaction, the optimized two-step heating profile is compared to products heated directly to the the higher annealing temperature. Vertical lines in each panel compare lattice parameters of the binary nitride (dash-dot, black: ZrN, NbN, Mo_2N) against the calculated ternary metal nitride lattice parameters (solid, calculated patterns in Fig. 1). Corresponding $MgCl_2$ (*) peaks labeled at 34.97 and 46.05 °2θ.

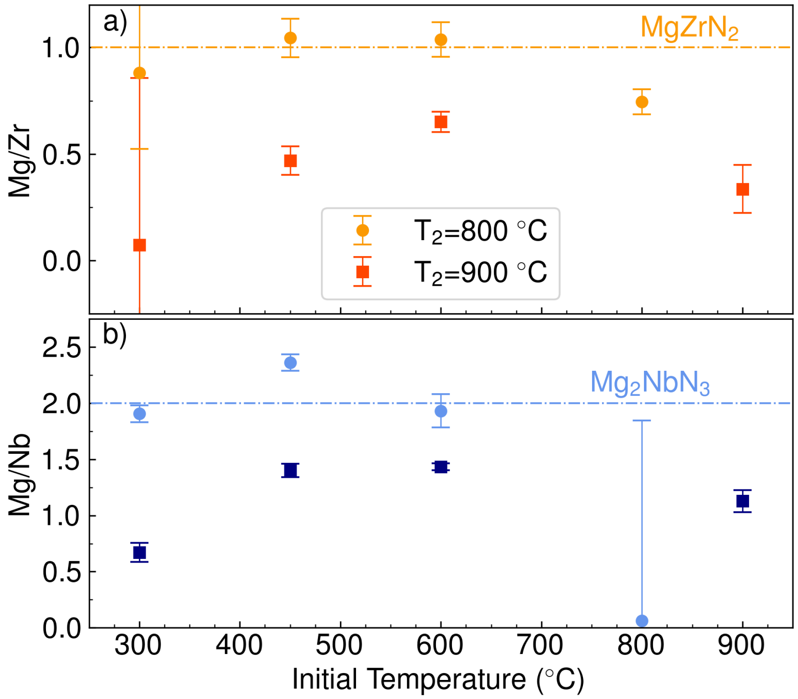

Figure S3: Calculated cation site occupancy values for rocksalt a) $MgZrN_2$ and b) Mg_2NbN_3 as a function of initial dwell temperature in the presented two-step reactions. The subsequent higher annealing temperature is denoted by a change in data point (800 °C: circle; 900 °C: square). Occupancy values on the 4a Wykoff cation site were calculated from Rietveld analysis of PXRD patterns of reaction products.

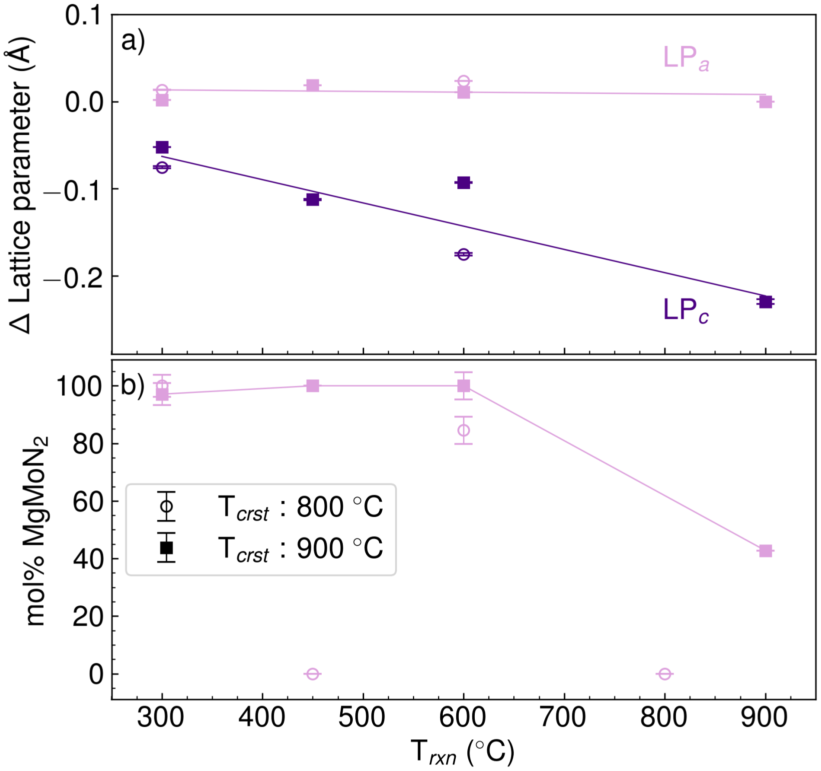

Figure S4: Analysis of the ternary nitride products as a function of the reaction temperature (T_{rxn}) and crystalization temperature (T_{crst}). Comparison of a) $MgMoN_2$ hexagonal lattice parameters, LP_a and LP_c and mole fraction of molybdenum nitride products, Mo_2N and $MgMoN_2$.

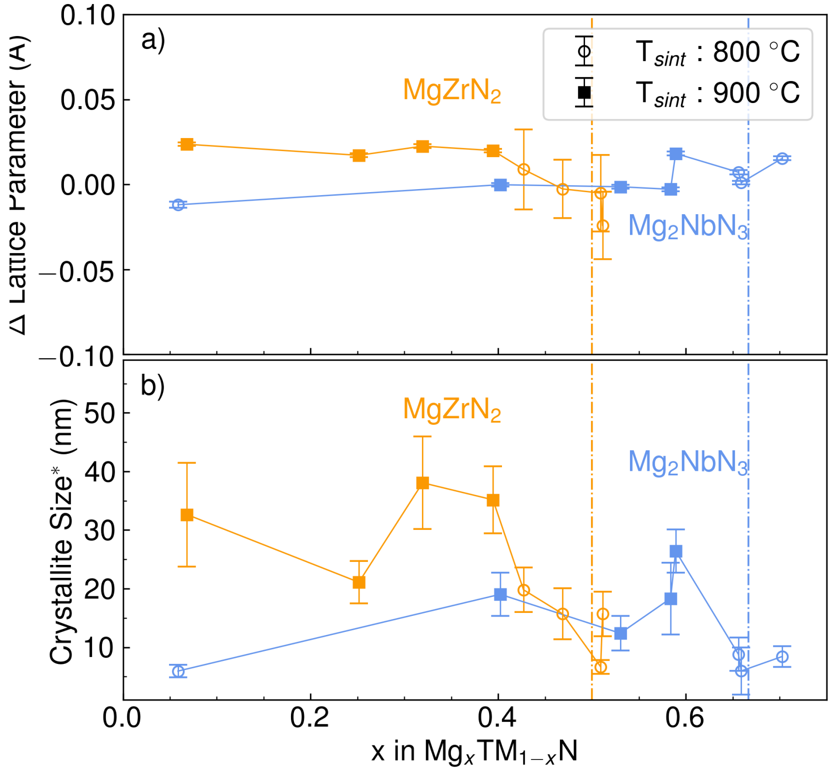

Figure S5: Analysis of the rocksalt ternary nitride products as a function of the reaction temperature (T_{rxn}) and crystalization temperature (T_{crst}). For rocksalt products in a), cubic lattice parameters of $Mg_xZr_{1\text{-}x}N$ and $Mg_xNb_{1\text{-}x}N$ are compared to b) the size of coherently scattering crystalline domains as calculated from PXRD data as compared to x in $Mg_xNb_{1\text{-}x}N$.
